# Supplementary material for: Efficient and scalable synthesis of highly aligned and compact two-dimensional nanosheet films with record performances
Source: Nat Commun. 2018 Aug 28;9:3484. doi: 10.1038/s41467-018-05723-2 (PMC6113301; doi:10.1038/s41467-018-05723-2)
Supplement: Supplementary file 1 — Supplementary Information [file 41467_2018_5723_MOESM1_ESM.pdf]

Efficient and scalable synthesis of highly aligned and compact two-dimensional nanosheets films with record performances

Zhong *et al*.

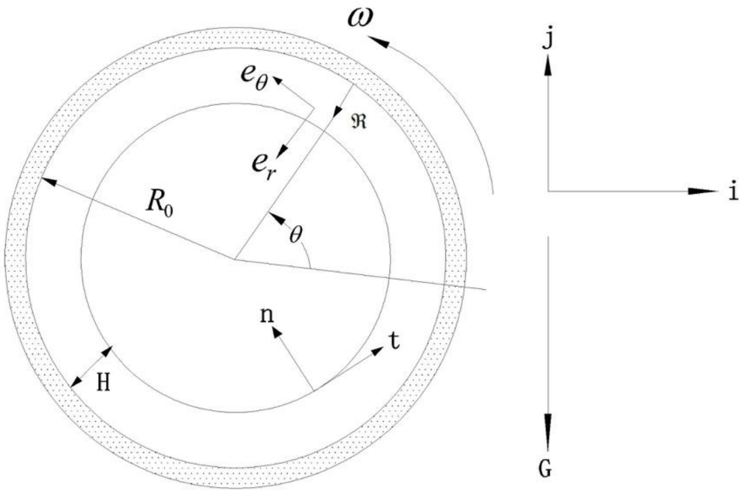

Supplementary Figure 1. **Schematic of a rotating hollow cylinder partially filled with a liquid layer.** The meanings of all the symbols are shown in Supplementary Note 1.

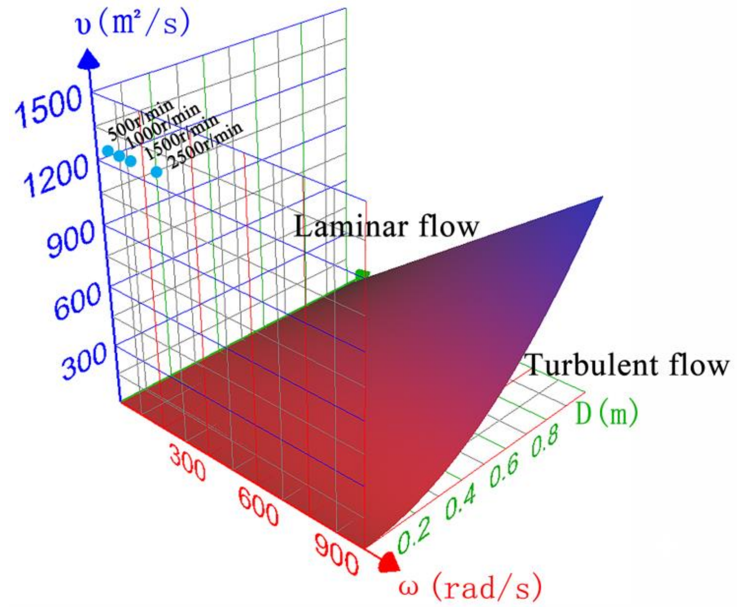

Supplementary Figure 2. **The phase diagram for the laminar and turbulent phase.** The bottom-right and up-left corresponds to the turbulent flow and laminar flow, respectively. The blue dots represent the experimental conditions (viscosity of 1.26 Pa.s, corresponding to the concentration of GO dispersion of ~ 8 mg mL^{-1}) that are used in our study. In fact, the real viscosity should be much higher than the original value because of the water evaporation caused by heating during the CCC process. Therefore, these blue dots should move further away from the phase transition interface.

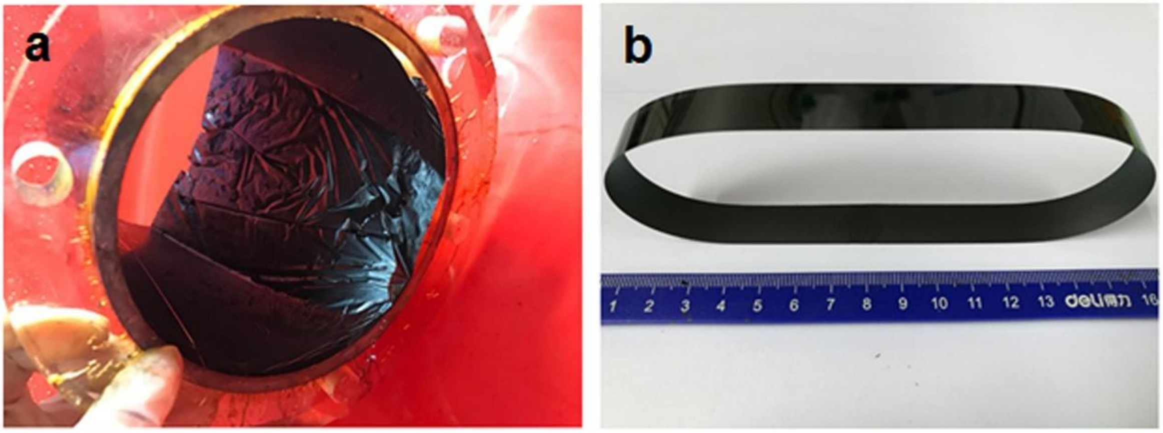

Supplementary Figure 3. **rGO films synthesized by CCC method. a**, A hydrophobic rGO film with part immersing into water, showing that it can be easily separated from the silica tube. **b**, A rGO bracelet directly cut out of a continuous rGO film and peeled off from the silica tube.

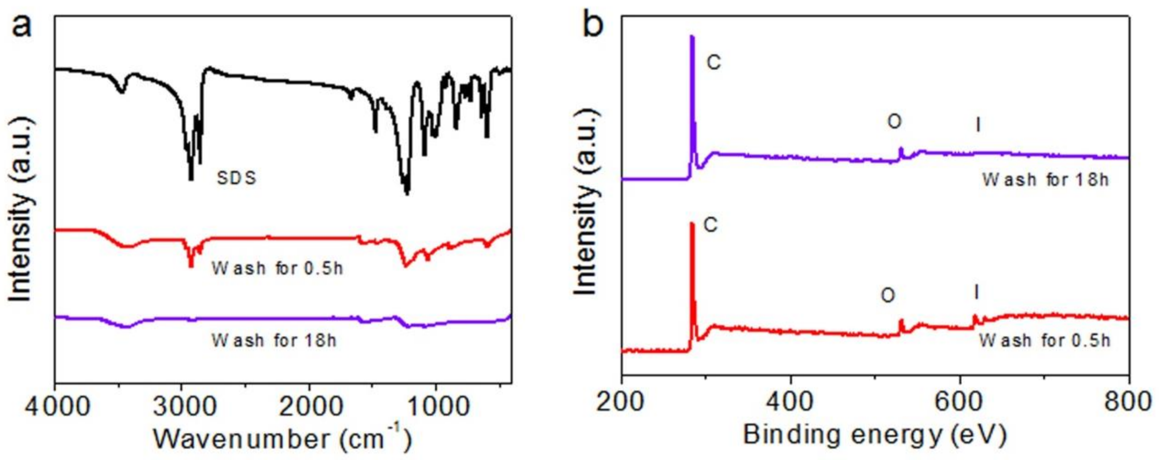

Supplementary Figure 4. **Characterization of the residual SDS and iodine in the rGO/SWCNT hybrid films.** FTIR (**a**) and XPS (**b**) spectra of the rGO/SWCNT hybrid film soaked in DI water for different time (0.5 h, 18 h) followed by ethanol washing for 2 h. The peaks at 2853 cm^{-1} and 2992 cm^{-1} correspond to the vibrational modes of methyl and methylene group in SDS, respectively, which were used to measure qualitatively the amount of residual SDS, and both of the two peaks diminish with increasing the soaking time in water.

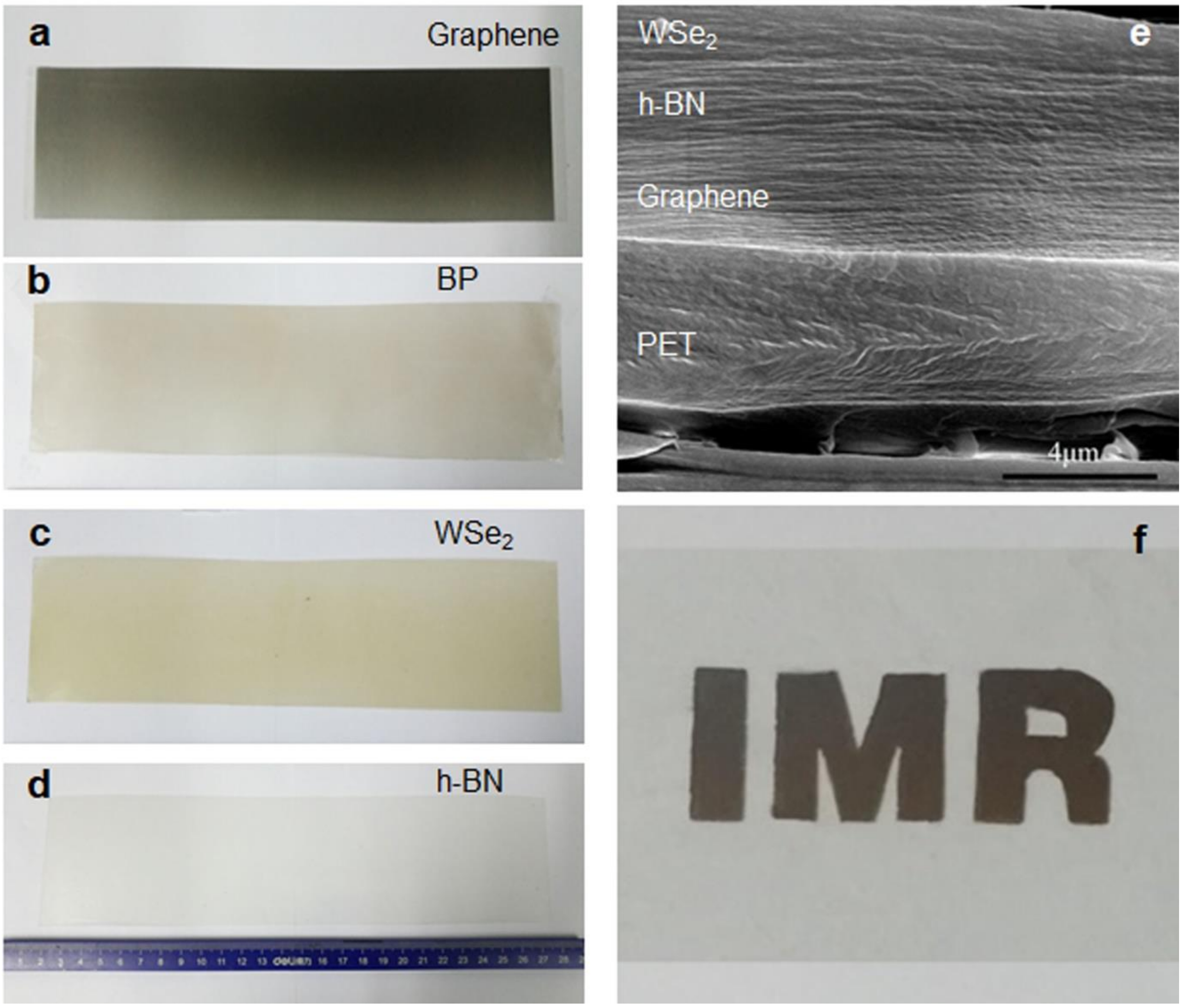

Supplementary Figure 5. **2D nanosheet films and heterostructures synthesized by CCC method on PET substrate. a**-**d**, Photos of a graphene film (**a**), black phosphorus (BP) film (**b**), WSe_2 film (**c**), and hexagonal boron nitride (h-BN) film (**d**). **e**, SEM image of a vertical graphene/h-BN/WSe_2 heterostructure film. **f**, Patterned graphene films.

Supplementary Table 1. Comparison of the GO films synthesized by different methods with different GO nanosheets.

Synthesis method	lateral size of GO nanosheets (μm)	C/O ratio of GO nanosheets	Interlayer Spacing (Å)	FWHM (degree)	Reference
VF	1-2	1.2	8.04	0.69	1
VF	1-2	1.2	8.05	0.55	1
VF	1-5	NA	8.03	2.06	2
VF	NA	NA	8.3	NA	3
VF	0.5	NA	7.96	1.71	4
VF	NA	NA	9.4	0.74	5
BCO	40	NA	9.07	0.75	6
SC	NA	0.96	8.6	1.71	7
SE	NA	1.49	7.01	0.95	8
SCO	44	NA	9.42	2.74	9
VS	1	2.38	8.50	2.01	10
VF	5 (5 ^{o}C)	2.25	7.90	1.09	11
VF	5 (35 ^{o}C)	2.26	7.5	0.54	11
VF	5	2.32	9.40	0.75	12
VF	33	2.4	9.50	0.95	12
VF	60	2.45	9.10	1.31	12
VF	93	2.61	8.58	0.86	12
CCC@2500	1	1.7	7.5	0.55	This work

VF = Vacuum filtration

NA = Not available

BCO = Bar coating

SC = Solvent casting

SE = Solvent evaporation

SCO = Spin coating

VS = Vacuum suction.

CCC = Continuous centrifugal casting

Supplementary Table 2. Structure, strength and electrical conductivity comparison of the rGO films synthesized by different methods.

Synthesis method	lateral size (μm)	XRD Peak (degree)	FWHM (degree)	Strength (MPa)	Conductivity (S cm^{-1})	Reference
VF	5	NA	NA	20	190	12
VF	33	NA	NA	24	380	
VF	60	NA	NA	26	480	
VF	93	NA	NA	28	500	
SA	5	24.4	1.31	162	298	13
VF	1.3	23.37	4.97	150	40	14
VF@220 ºC	1.3	25.66	1.7	293	120	
VF@500 ºC	1.3	26.15	1.55	90	295	
SE	2.5	NA	NA	117	45	15
ESD	NA	24.12	3.5	66	26	16
SA	22	24.62	3.56	170	210	17
VF	NA	NA	NA	118	50	18
CCC@1500	20	25.7	1.13	591	602	This work
CCC@2500	20	25.94	1.02	662	647	

SA = Self assembly

ESD = electro-spray deposition

Supplementary Table 3. Capacitance comparison of the all-solid-supercapacitors based on different electrode materials.

Electrode material	Specific capacitance ($F\ g^{-1}$)	Volumetric capacitance ($F\ cm^{-3}$)	Discharge rate	Reference
Graphene/CNT	508	300	26.7 $mA\ cm^{-3}$	19
Graphene/CNT	~237	158	0.1 $mA\ cm^{-2}$	20
MWCNT/PEDOT	119	179	10 $mV\ s^{-1}$	21
CF/MnO_2	1	2.5	20 $mA\ cm^{-3}$	22
Co_3O_4@Ni Wire/Graphene	0.3	2.1	20 $mA\ cm^{-3}$	23
CF@ZnO@$MnO_2$$^{+}$	<0.64	0.325	50 $mA\ cm^{-3}$	24
CNT/MnO_2 Yarn^{++}	<17	25.4	10 $mV\ s^{-1}$	25
N-doped CF	260	4.3	500 $mA\ cm^{-3}$	26
B, N co-doped graphene aerogels	62	49.6	5 $mV\ s^{-1}$	27
Undoped graphene aerogels	43	34.4	5 $mV\ s^{-1}$	27
N-doped graphene aerogels	52	41.6	5 $mV\ s^{-1}$	27
B-doped graphene aerogels	55	44	5 $mV\ s^{-1}$	27
Graphene paper^{+++}	29	23.2	5 $mV\ s^{-1}$	27
Laser scribing graphene	202	0.45	1 $A\ g^{-1}$	28
Activated CC	0.003	0.0088	10 $mV\ s^{-1}$	29
Coaxial fiber of aligned CNT fiber and sheet	59	32.09	46 $mA\ cm^{-3}$	30
GF@3D-G fiber	40	9.2	142 $\mu A\ cm^{-2}$	31
CF@ZnO@MnO_2*	0.033	0.066	100 $mV\ s^{-1}$	32
CF@MnO_2/CF@Fe_2O_3	91.3	1.5	2 $mA\ cm^{-2}$	33
N-doped CF^{++++}	77.4	38.7	25 $mV\ s^{-1}$	34
Graphene hydrogel	187	31	10 $mV\ s^{-1}$	35
rGO/SWCNT	255	407	114 $mA\ cm^{-3}$	This work

+ The author did not provide the specific capacitance directly, which was estimated by assuming the density of the electrode as 2 g cm^{-3}.

++The specific capacitance was estimated by assuming the density of the electrode as 1.5 g cm^{-3}.

+++ The volumetric capacitance was estimated by assuming the density of the electrode as 0.8 g cm^{-3}.

++++ The volumetric capacitance was estimated by assuming the density of the electrode as 0.1 g cm^{-3}.

* The author did not provide the specific capacitance directly, which was estimated by the given geometrical size and density of the plastic wire by 2 g cm^{-3}.

Supplementary Note 1. Analysis of fluid dynamics during CCC process

In a liquid flow, shear stress is generated when there is velocity gradient in the direction that is perpendicular to the velocity direction. In our method, when the hollow tube is rotated, the first liquid layer that is adjacent to the tube inner surface will stick on the tube according to the non-slippery boundary condition, and thus has the same linear velocity with the inner surface of the rotating tube. The second liquid layer is then pulled by the first liquid layer to move along because of the viscosity of the liquid. Such disturbation generated by the tube rotation thus propagates from the liquid-solid interface (tube inner surface) deep into the liquid flow. The transient shear rate distribution at the beginning of the tube rotation (at 1 microsecond) was investigated by the software of CONSOL. As shown in Fig. 1b in the main text, the disturbation transports from the liquid-solid boundary into liquid immediately once the tube starts to rotate, generating uniform shear rate around the tube inner surface.

As discussed in the main text, the liquid layer around the inner surface of a rotating hollow tube, typically considered as rimming flow, is a non-trivial example of a steady, two-dimensional, viscous flow with a free surface. As shown in Supplementary Fig. 1, we considered a horizontally placed circular hollow cylinder with inside radius R_0, which rotates at a constant angular velocity (ω) around its axis. Polar coordinates were used: Θ and $\Re$ are the angular azimuthal coordinate and radial coordinate, respectively, and $\mathbf{e}_\theta$ and $\mathbf{e}_r$ are the corresponding unit tangent vectors. The cylinder is partially filled with Newtonian liquid which is distributed in a layer around the inner wall. Strictly speaking, the thickness of the liquid layer varies with the cylinder and is a function of θ, that is $H(\theta)$, because of the influence of the local gravitational acceleration (which is denoted as $\mathbf{G}$ along the vertical direction of $\mathbf{j}$). Since the gas occupying the rest of the volume in the cylinder is inviscid, the viscous stress exerted by the relatively rarefied gas at the gas/liquid interface is negligible, and thus can be modeled as by a uniform pressure $\mathbf{P}_a$.

Based on the above analysis, this trimming flow can be modeled mathematically as follows:

$$\left\{\begin{matrix} \mathbf{V}\cdot\nabla\mathbf{V} = -\rho^{-1}\nabla\mathbf{P} + \upsilon\nabla^2\mathbf{V} - \mathrm{G}\mathbf{j} \\ \nabla\cdot\mathbf{V} = 0. \end{matrix}\right\} \quad (1)$$

where $\mathbf{V}(R)$ and $\mathbf{P}(R)$ are liquid velocity and pressure fields, respectively. The density ρ, viscosity μ, kinematic viscosity $\nu=\mu/\rho$ and surface tension σ of the liquid are all assumed uniform.

The no-slip boundary condition at the liquid-wall interface is:

$$\mathbf{V}=\omega R_0\mathbf{e}_\theta \quad \text{at} \quad \mathbf{R}= -R_0\mathbf{e}_\mathrm{r} \quad (2)$$

The balance of traction at the liquid/gas interface gives the boundary condition:

$$(P-P_a)\mathbf{n} - \mu\left[\nabla\mathbf{V} + (\nabla\mathbf{V})^\mathrm{T}\right]\cdot\mathbf{n} + 2\sigma K\mathbf{n} = 0 \quad at \quad \mathbf{R} = -(R_0 - H)\boldsymbol{e}_\mathrm{r}. \quad (3)$$

where $\mathbf{n}$ is the unit normal to the interface, directed to the gas, and K is the mean curvature of the interface:

$$\mathrm{K} = \frac{1+2[H_\theta/(R_0-H]^2 + H_{\theta\theta}/(R_0-H)}{2(R_0-H)\{1+[H_\theta/(R_0-H)]^2\}^{\frac{3}{2}}} \quad (4)$$

Since no liquid can cross the interface, the velocity along the normal direction should be zero:

$$\mathbf{V} = \omega R_0\mathbf{e}_\theta \quad \text{at} \quad \mathbf{R} = -R_0\mathbf{e}_\mathrm{r} \quad (5)$$

It is obvious that the solution for the above equations must be periodic with respect to the coordinate of θ with a period of 2π.

The average thickness of the liquid layer (D) is given by

$$2R_0D - D^2 = \frac{1}{2\pi}\int_0^{2\pi}(2R_0H - H^2)dX \quad (6)$$

For the high rotation rate, G can be neglected, and thus we assume $G = 0$.

An analytical solution can be obtained:

$$\left.\begin{matrix} \mathbf{V} = \omega(R_0 - Y)\mathbf{e}_\theta, \\ P - P_a = \rho\,\Omega^2\left[R_0(D-Y) - \frac{1}{2}(D^2 - Y^2)\right] - \frac{\sigma}{R_0 - D}, \\ H = D. \end{matrix}\right\} \quad (7)$$

The derived shear stress for this Newtonian flow is:

$$\boldsymbol{\sigma}_{\mathrm{shear}} = \mu\omega\mathbf{e}_{\theta} \quad (8)$$

During the continuous centrifugal casting process, the 2D nanosheets are also subjected to centrifugal force at the same time. The centrifugal stress that is applied on 2D nanosheets is given by:

$$\boldsymbol{\sigma}_{\mathrm{centrifugal}} = \rho_g t_g R_0 \omega^2 \mathbf{e}_{\mathrm{r}} \quad (9)$$

where ρ_g and t_g are the density and thickness of 2D nanosheets, respectively.

Supplementary Note 2: Discussion on the data shown in Supplementary Table 1

Dip coating, spin coating, and spray coating were also reported for the synthesis of GO film, but the films obtained are mostly used for transparent conductive films and the XRD data were not provided. In Supplementary Ref. 11, authors synthesized GO at two different temperatures (5 ^{o}C and 35 ^{o}C) to control the defects. The GO nanosheets synthesized at 5 ºC show much less defects than those synthesized at 35 ºC. As shown in Supplementary Ref. 12, the lateral size of GO nanosheets plays an important role in the interlayer spacing and alignment of the films. The relatively small interlayer spacing shown in Supplementary Ref. 8 is probably due to the large lateral size of the GO nanosheets and the annealing at 80 ^{o}C for 8 hours, and the relatively small FWHM shown in Supplementary Ref. 11 is probably due to the large lateral size of the GO nanosheets as well.

Supplementary References

1. Yeh, C. N., Raidongia, K., Shao, J., Yang, Q. H. & Huang, J. X. On the origin of the stability of graphene oxide membrane in water. *Nat. Chem.* **7**, 166–170 (2015).
2. Joshi, R. K., Carbone, P. & Wang, F. C. Precise and ultrafast molecular sieving through graphene oxide membranes. *Science* **343**, 752–754 (2014).
3. Dikin, D. A. *et al.* Preparation and characterization of graphene oxide paper. *Nature* **448**, 457–460 (2007).
4. Li, H. *et al.* Ultrathin, molecular sieving graphene oxide membranes for selective hydrogen separation. *Science* **342**, 95–98 (2013).
5. Tian, T., Cao, Y., Wang, Y., Yang, M. & Feng, J. Realizing ultrahigh modulus and high strength of macroscopic graphene oxide papers through crosslinking of mussel inspired polymers. *Adv. Mater.* **4**, 2980–2983 (2013).
6. Cruz-Silva, R. *et al.* Super-stretchable graphene oxide macroscopic fibers with outstanding knotability fabricated by dry film scrolling. *ACS Nano* **8**, 5959–5967 (2014).
7. Moon, I. K., Lee, J., Ruoff, R. S. & Lee, H. Reduced graphene oxide by chemical graphitization. *Nat. Commun.* **1**, 73 (2010).
8. Chen, C. M. *et al.* Annealing a graphene oxide film to produce a free standing high conductive graphene film. *Carbon* **50**, 659–667 (2012).
9. Luo, Z. T., Lu, Y., Somers, L. A. & Johnson, A. T. High yield preparation of macroscopic graphene oxide membranes. *J. Am. Chem. Soc.* **131**, 898–899 (2009).
10. Huang, K. *et al.* A graphene oxide membrane with highly selective molecular separation of aqueous organic solution. *Angew. Chem. Int. Ed.* **53**, 6929-6932 (2014).
11. Zhang, M. *et al.* Multifunctional pristine chemically modified graphene films as strong as stainless steel. *Adv. Mater.* **42**, 6708–6713 (2015).
12. Lin, X. *et al.* Fabrication of highly-aligned, conductive, and strong graphene papers using

ultralarge graphene oxide sheets. *ACS Nano* **6**, 10708–10719 (2012).

13. Pei, S., Zhao, J. P., Du, J. H., Ren, W. C. & Cheng, H. M. Direct reduction of graphene oxide films into highly conductive and flexible graphene films by hydrohalic acids. *Carbon* **48**, 4466–4474 (2010).
14. Chen, H., Muller, M. B., Gilmore, K. J., Wallace, G. C. & Li, D. Mechanically strong, electrically conductive, and biocompatible graphene paper. *Adv. Mater*. **20**, 3557–3561 (2008).
15. Cui, W. et al. A strong integrated strength and toughness artificial nacre based on dopamine cross-linked graphene oxide. *ACS Nano* **8**, 9511–9517 (2014).
16. Xin, G. Q. *et al.* Large area freestanding graphene paper for superior thermal management. *Adv. Mater*. **26**, 4521–4526 (2014).
17. Liu, Z. *et al.* Wet spun continuous graphene film. *Chem. Mater*. **26**, 6786–6795 (2014).
18. Cheng, Q. F. *et al.* Ultratough artificial nacre based on conjugated cross-linked graphene oxide. *Angew. Chem*. *-Int. Edit.* **25**, 3750–3755 (2013).
19. Yu, D. S. *et al.* Scalable synthesis of hierarchically structured carbon nanotube-graphene fibres for capacitive energy storage. *Nat. Nanotechnol*. **9**, 555–562 (2014).
20. Kou, L. *et al.* Coaxial wet spun yarn supercapacitors for high energy density and safe wearable electronics. *Nat. Commun*. **3**, 3754 (2014).
21. Lee, J. A. *et al.* Ultrafast charge and discharge biscrolled yarn supercapacitors for textiles and microdevices. *Nat. Commun*. **4**, 1970 (2013).
22. Xiao, X. *et al.* Fibre based all solid state flexible supercapacitors for self-powered system. *ACS Nano* **6**, 9200–9206 (2012).
23. Wang, X. *et al.* Fiber - based flexible all - solid - state asymmetric supercapacitors for integrated photodetecting system. *Angew. Chem. Int. Ed*. **126**, 1880–1884 (2014).
24. Yang, P. H. *et al.* Hydrogenated ZnO core-shell nanocables for flexible supercapacitors

and self-powered systems. *ACS Nano* **7**, 2617–2626 (2013).

25. Choi, C. *et al.* Flexible supercapacitor made of carbon nanotube yarn with internal pores. *Adv. Mater*. **26**, 2059–2065 (2014).
26. Miao, F., Shao, C. L. & Li, X. H. Flexible solid-state supercapacitors based on freestanding nitrogen-doped porous carbon nanofibers derived from electrospun polyacrylonitrile@polyaniline nanofibers. *J. Mater. Chem. A*. **4**, 4180–4187 (2016).
27. Wu, Z. S. *et al.* Three-Dimensional nitrogen and boron co-doped graphene for high performance all solid state supercapacitors. *Adv. Mater*. **24**, 5130–5135 (2012).
28. El-Kady, M. F., Strong, V., Dubin, S. & Kaner, R. B. Laser scribing of high-performance and flexible graphene-based electrochemical capacitors. *Science* **335**, 1326–1330 (2012).
29. Wang, G. M. *et al.* Solid state supercapacitor based on activated carbon cloths exhibits excellent rate capability. *Adv. Mater*. **26**, 2676–2682 (2014).
30. Chen, X. L. *et al.* Novel electric double layer capacitor with a coaxial fiber structure. *Adv. Mater*. **26**, 6436–6441 (2013).
31. Meng, Y. N. *et al.* All graphene core sheath microfibers for all solid state, stretchable fibriform supercapacitors and wearable electronic textiles. *Adv. Mater*. **25**, 2326–2331 (2013).
32. Bae, J. *et al.* Fiber Supercapacitors Made of Nanowire – Fiber Hybrid Structures for Wearable/Flexible Energy Storage. *Angew. Chem. Int. Ed*. **50**, 1683–1687 (2011).
33. Yang, P. H. *et al.* Low cost high performance solid state asymmetric supercapacitors based on MnO_2 nanowires and Fe_2O_3 nanotubes. *Nano Lett*. **14**, 731–736 (2014)
34. Chen, L. F., Huang, Z. H. & Liang, H. W. Flexible all solid state high power supercapacitor fabricated with nitrogen doped carbon nanofiber electrode material derived from bacterial cellulose. *Energy Environ. Sci*. **6**, 3331–3338 (2013).
35. Xu, Y. X. *et al.* Flexible solid state supercapacitors based on three dimensional graphene

hydrogel films. *ACS Nano* **7**, 4042–4049 (2013).
